# Identification of Novel Single-Nucleotide Variants With Potential of Mediating Malfunction of MicroRNA in Congenital Heart Disease

**DOI:** 10.3389/fcvm.2021.739598

**Published:** 2021-09-10

**Authors:** Wangkai Liu, Liangping Cheng, Ken Chen, Jialing Wu, Rui Peng, Yan-Lai Tang, Jinghai Chen, Yuedong Yang, Peiqiang Li, Zhan-Peng Huang

**Affiliations:** ^1^Department of Pediatrics, The First Affiliated Hospital, Sun Yat-sen University, Guangzhou, China; ^2^Department of Cardiology, Center for Translational Medicine, Institute of Precision Medicine, The First Affiliated Hospital, Sun Yat-sen University, Guangzhou, China; ^3^School of Data and Computer Science, Sun Yat-sen University, Guangzhou, China; ^4^Obstetrics and Gynecology Hospital, Institute of Reproduction and Development, Fudan University, Shanghai, China; ^5^Department of Cardiology, Provincial Key Lab of Cardiovascular Research, Second Affiliated Hospital, Institute of Translational Medicine, Zhejiang University School of Medicine, Hangzhou, China; ^6^Key Laboratory of Machine Intelligence and Advanced Computing, Sun Yat-sen University, Ministry of Education, Guangzhou, China; ^7^Institute of Genetics, School of Basic Medical Sciences, Lanzhou University, Lanzhou, China; ^8^NHC Key Laboratory of Assisted Circulation, Sun Yat-sen University, Guangzhou, China; ^9^National-Guangdong Joint Engineering Laboratory for Diagnosis and Treatment of Vascular Diseases, Guangzhou, China

**Keywords:** neural crest cells, single nucleotide variant, congenital heart defect, microRNA, post-transcriptional regulation

## Abstract

Congenital heart defects (CHDs) represent the most common human birth defects. Our previous study indicates that the malfunction of microRNAs (miRNAs) in cardiac neural crest cells (NCCs), which contribute to the development of the heart and the connected great vessels, is likely linked to the pathogenesis of human CHDs. In this study, we attempt to further search for causative single-nucleotide variants (SNVs) from CHD patients that mediate the mis-regulating of miRNAs on their downstream target genes in the pathogenesis of CHDs. As a result, a total of 2,925 3′UTR SNVs were detected from a CHD cohort. In parallel, we profiled the expression of miRNAs in cardiac NCCs and found 201 expressed miRNAs. A combined analysis with these data further identified three 3′UTR SNVs, including NFATC1 c.^*^654C>T, FGFRL1 c.^*^414C>T, and CTNNB1 c.^*^729_^*^730insT, which result in the malfunction of miRNA-mediated gene regulation. The dysregulations were further validated experimentally. Therefore, our study indicates that miRNA-mediated gene dysregulation in cardiac NCCs could be an important etiology of congenital heart disease, which could lead to a new direction of diagnostic and therapeutic investigation on congenital heart disease.

## Introduction

Congenital heart defects (CHDs) represent the most common human birth defects. Among the spectrum of CHDs, malformations in the aortic arch artery and outflow tract comprise nearly 40% of CHDs, and ventricular septal defect is estimated to account for about 30% of CHDs. Previous studies have demonstrated that cardiac neural crest cells (NCCs), which migrate from the dorsal neural tube of the embryo, contribute to the development of the ventricular septum and great arteries connected to the heart (1, 2).

Neural crest cells are a transient, migratory population of cells that give rise to myriad derivatives. Neural crest progenitors lie in the neural tube border. After the closure of the neural tube, these cells leave the neural tube and migrate throughout the body along the dorsoventral axis. They can differentiate into many cell types, including cardiac smooth muscle cells, chondrocytes, melanocytes, and neurons (3). Cranial NCCs, which originate from the dorsal neural tube between the middiencephalon and the caudal limit of somite 5, give rise to cranial ganglia, the maxilla, the mandible, and other structures such as muscle and cartilage of the head and neck (4). There is also a subset of NCCs defined as cardiac NCCs, which arise from the dorsal neural tube between the midotic placode and the caudal limit of somite 3. They migrate into pharyngeal arches 3, 4, and 6 (1). Cardiac NCCs populate the aorticpulmonary septum and conotruncal cushion and contribute to the smooth muscle of great vessels. Studies have revealed that several factors, including Wnt (5), Fgf (6), Tgf-β (7), BMP (8) families, and Shh (9), and their signaling pathways are critical to the development of neural crest derivatives. Disruption of these signaling pathways in NCCs in a mouse model leads to multiple developmental defects, which assemble CHDs found in human patients.

MicroRNAs (miRNAs), a large set of small non-coding RNAs, have been discovered in both animals and plants. These small RNAs post-transcriptionally regulate gene expression by destabilization and degradation of mRNA or translational repression (10). Previous studies from our lab and other groups have revealed that miRNAs play important roles in cell proliferation, differentiation, migration, and apoptosis during proper development and diseases (11–13). More importantly, our previous study demonstrated that globe disruption of the expression of miRNAs in NCCs resulted in a spectrum of CHDs in a mouse model (14), which indicates miRNAs could be involved in the pathogenesis of human CHDs. However, how miRNAs connect to CHDs in human patients remains largely unknown.

In this study, we searched for single-nucleotide variants (SNVs) from the 3′UTR region of genes, which could be associated with the CHD through a miRNA regulating mechanism, from a cohort of CHD. Combined with the profiling of miRNA expression in cardiac NCCs, we identified four novel SNVs with potential of interfering with miRNA-mediated gene dysregulation from 2,925 3′UTR SNVs in human patients. Experimental evidences support three out of four predictive miRNA-mediated mis-regulating. Therefore, our data suggest that the malfunction of miRNA is a new direction of mechanistic study and clinical application for congenital heart disease.

## Materials and Methods

### Paired-End Sequencing for Detecting Single-Nucleotide Variants From Patients

Probes were designed to capture open reading frame (ORF), 3′UTR, 5′UTR, and 2KB of upstream promotor of selected genes on Agilent eArray platform against hg19 human genome. Genomic DNA from patients were sonicated into fragments. A DNA library from an individual was barcoded and used for generating the library for paired-end sequencing according to manufacturers' instruction for SureSelect XT Target Enrichment System for Illumina Paired-End Sequencing Library (Agilent). Reads were aligned in the reference genome with Burrows–Wheeler Aligner after filtering low quality reads. Single-nucleotide variants were then identified according to a previous report (15).

### Annotating Variants

To obtain the information of SNVs residing in the transcripts, we employed ANNOVAR (version 2018-04-16) to annotate them against the refSeq gene annotation database (hg19_refGene.txt) (16). It should be noted that the deleted/inserted nucleotides in deletions/insertions could not be processed by ANNOVAR directly. We thereby manually converted them into 1-bp insertions or deletions by adding or deleting one more nucleotide after the position of variants to make them compatible with ANNOVAR. In this way, the gene and transcript annotations for all the variants were obtained.

### Identifying Single-Nucleotide Variants in miRNA Binding Sites

We first obtained miRNA-transcript pairs predicted by the state-of-the-art MirTarget model (v4.0) from the miRDB database (17) to find which variants reside in the binding sites of miRNAs. Notably, the miRDB database only provides the predicted miRNA-transcript pairs. To solve the problem of lacking the exact location of binding sites of miRNAs, we identified the candidate binding sites based on the rule that canonical binding sites are reverse complementary to the second to eighth nucleotides in miRNA sequences (18, 19). The sequences of miRNAs were obtained from the miRBase database (20), and the sequences of transcripts (mature mRNA sequences) were extracted from the GENCODE GRCh37 reference genome (21).

### MicroRNA Profiling With Mouse Embryonic Cardiac NCCs and Non-NCCs

Wnt1-Cre mice were crossed with Rosa-mTmG reporter mice to obtain green fluorescent protein (GFP)-labeled offspring. Green fluorescent protein-labeled embryonic day 10.5 embryos were collected for cardiac NCCs collection. Cardiac NCC-derived third, fourth, and sixth pharyngeal arches and outflow were dissected out and digested with collagenase. Single cells after digestion were subjected to fluorescence-activated cell sorting (FACS) on a MoFlo cell sorter. Some 5–10 × 10^5^ GFP-positive NCCs and non-NCCs were obtained to yield 5–10 μg total RNA for further experiments. Total RNAs were then subjected to detection of global miRNA expression with Affymetrix microRNA microarray (miRNA 1.0) according to the manufacturer's protocol. Signals were obtained with Scanner 3000 Autoloader and analyzed with Expression Console^TM^ software.

### Confirmation of SNVs With Singer Sequencing

To confirm the SNVs detected from the paired-end sequencing, primers were designed as follows for polymerase chain reaction (PCR) reaction to amplify the SNV-contained DNA fragments from patients: NFATC1-F: 5′-ACCCTCTGTATGAATGAAGAGA-3′; NFATC1-R: 5′-GCCTAGATATGTACACACATGC-3′; FGFRL1-F: 5′-ACACAGATAAGCTGCCCAAATG-3′; FGFRL1-R: 5′-AAGGCAGCATTATCTGTGTGTC-3′; CTNNB1-F: 5′-AAGCAGGTGGATCTATTTCATG-3′; CTNNB1-R: 5′-TTACTTACCACCCTCACAAACC-3′; SMAD2-F: 5′-GTTAAAAGTAACATTCTGGGCC-3′; SMAD2-R: 5′-CCAGAAATACCTTAAACGTGTT-3′. Polymerase chain reaction products were then purified for Singer sequencing to confirm the SNV.

### Constructs, Cell Culture, and Luciferase Reporter Assays

HEK293T cells were cultured in Dulbecco's modified essential medium (DMEM) supplemented with 10% fetal bovine serum (FBS) in a 5% CO_2_ atmosphere at 37°C. For construction of the 3′UTR-luciferase reporter, the multiple cloning site of the pGL3-Control vector (Promega) was removed and placed downstream of the luciferase gene. Short 3′UTR fragments of human NFATC1, FGFRL1, CTNNB1, and SMAD2 genes containing predicted miRNA binding sites were cloned into the modified pGL3-Control vector, resulting in the constructs Luc-NFATC1-WT-UTR, Luc-FGFRL1-WT-UTR, Luc-CTNNB1-WT-UTR, and Luc-SMAD2-WT-UTR. Similarly, mutant 3′UTR fragments harboring SNVs detected in patients were cloned into the modified pGL3-Control vector, resulting in mutant constructs. Luciferase reporter, Renilla control luciferase reporter, and miRNA mimic were transfected into HEK293T cells with Lipofectamine (Invitrogen) reagents according to manufacturers' instruction. Twenty-four hours after transfection, cell extracts were prepared, and luciferase activity was determined with dual luciferase assay kit (Promega). For luciferase assay, normalized Firefly luciferase activity from triplicate samples in 12-well plates relative to Renilla luciferase activity was calculated.

### Statistics

Values are reported as means ± SD unless indicated otherwise. The two-tailed Mann–Whitney U-test was used for two-group comparisons. Values of *p* < 0.05 were considered statistically significant.

### Data Deposit

Genome-wide raw data in this study were deposited in the Gene Expression Omnibus (GEO) database of the National Center for Biotechnology Information (NCBI) (Series GSE178823).

### Ethics Statement

All animal procedures were approved by the medical ethics committee of the First Affiliated Hospital, Sun Yat-sen University. All study protocols related to human patients were reviewed and approved by the local medical Ethics Committee. All individuals involved in the study have signed informed consent.

## Results

### Detecting 2,925 Single-Nucleotide Variants in 3′UTR of Genes From a CHD Cohort

**Figure 1:**
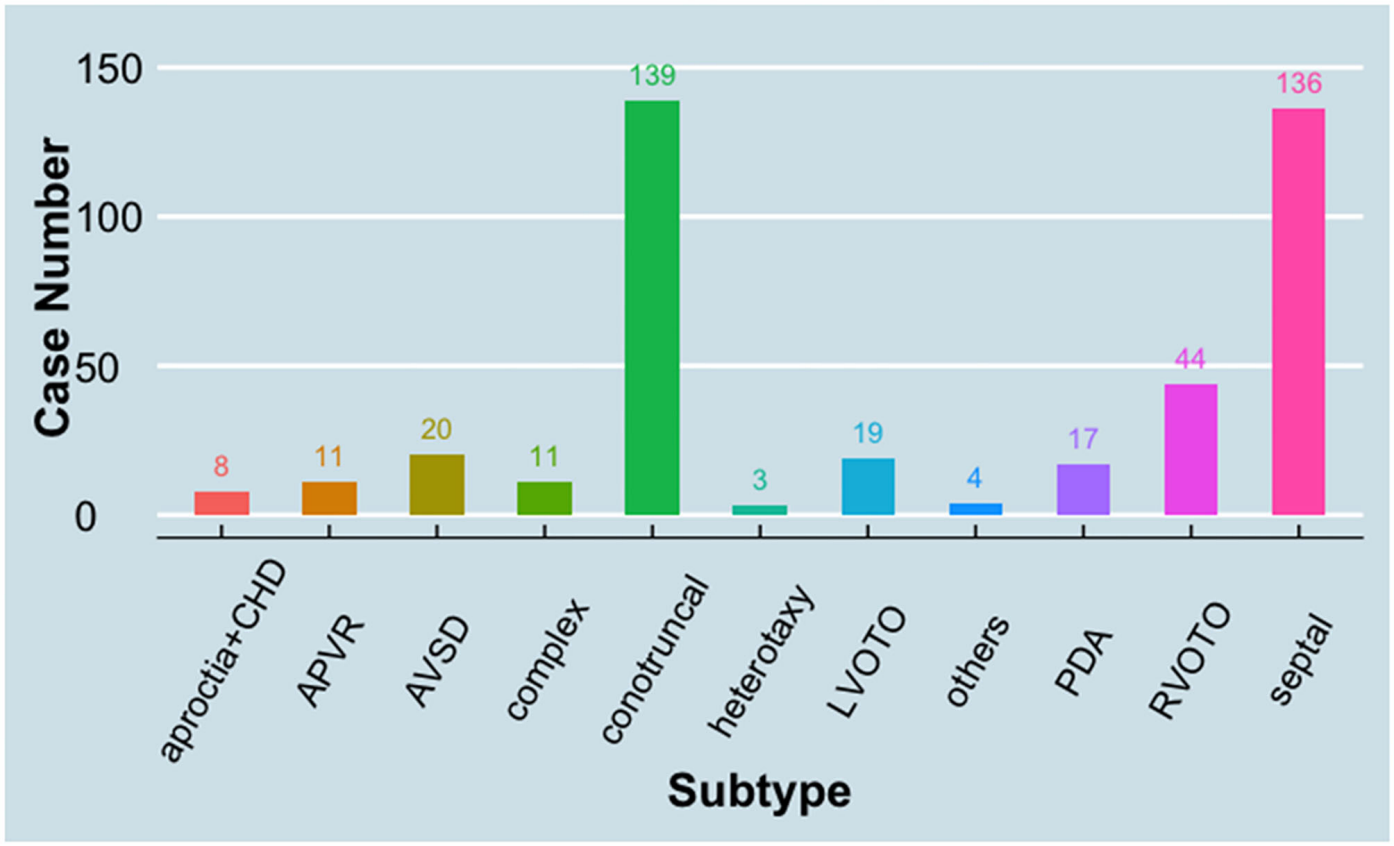
Distribution of case subtypes in the study. APVR, anomalous pulmonary venous return; AVSD, atrioventricular septal defect; CHD, congenital heart defect; LVOTO, left ventricular outflow tract obstruction; PDA, patent ductus arteriosus; RVOTO, right ventricular outflow tract obstruction.

A cohort of CHDs, including 412 patients (case group) and 213 healthy controls (control group), were enrolled between August 2008 and February 2011 in Shanghai and Shandong, China (22). The average age of the case group vs. control is 2.9 ± 2.7 vs. 7.1 ± 3.7 years. The gender distribution of the case group vs. control group is 231 males and 186 females vs. 106 males and 107 females (*p* > 0.05). The congenital defects were classified into 11 groups according to a previous report (23) (Figure 1). Among these groups, conotruncal defect, septal defect, and right ventricular outflow tract obstruction (RVOTO) represent the majority of CHD cases and account for 33.7, 33.0, and 10.7% of the total cases, respectively. There is no family history for CHD in all cases. All patients possess non-comprehensive CHDs, except eight cases of heart defects combined with aproctia.

In order to detect SNVs from CHD patients, a total of 252 genes important for cardiac development were selected for detection of single-nucleotide polymorphisms (SNPs), including 149 genes of signaling transduction (WNT, TGFβ, Notch, ERBB, FGF, and Cilia-Hedgehog), 40 genes of transcriptional factors, 46 genes related to “Folate and one carbon metabolism,” and 17 genes of structural proteins. Genomic DNA was chopped, and the regions of ORF, 3′UTR, 5′UTR, and 2KB of upstream promotor were captured by designed probes for paired-end sequencing (22). The sequencing yielded a set of data with average 38.1× coverage of targeted regions (38.7× in the control group vs. 38.0× in the case group). Targeted regions, which have an at least 4× coverage, is over 90% average in all samples (91.1% in control group vs. 91.6% in case group). As a result, 13,786 SNVs were identified in the analyses, including 11,486 SNVs in the case group and 5,870 SNVs in the control group. It is worthy to note that CHD patients have more rare SNVs, whose minor allele frequency (MAF) is <1%, than healthy controls. In detail, 66.2% (7,607) SNVs found in CHD patients are rare SNVs, while only 35.3% (2,072) SNVs found in healthy controls are rare ones. These data indicate that DNA mutation is a main contributor of pathogenesis of CHDs in the cohort.

In this study, we aim to explore the potential SNVs in 3′UTR interfering with the regulation between microRNA (miRNA) and mRNA in the pathogenesis of CHD. Therefore, a total of 2,925 SNVs located in 3′UTR of selected genes were obtained from the above identification and subjected to further analyses below.

### Profiling MicroRNA Expression in Migrating Neural Crest Cells From Embryo

**Figure 2:**
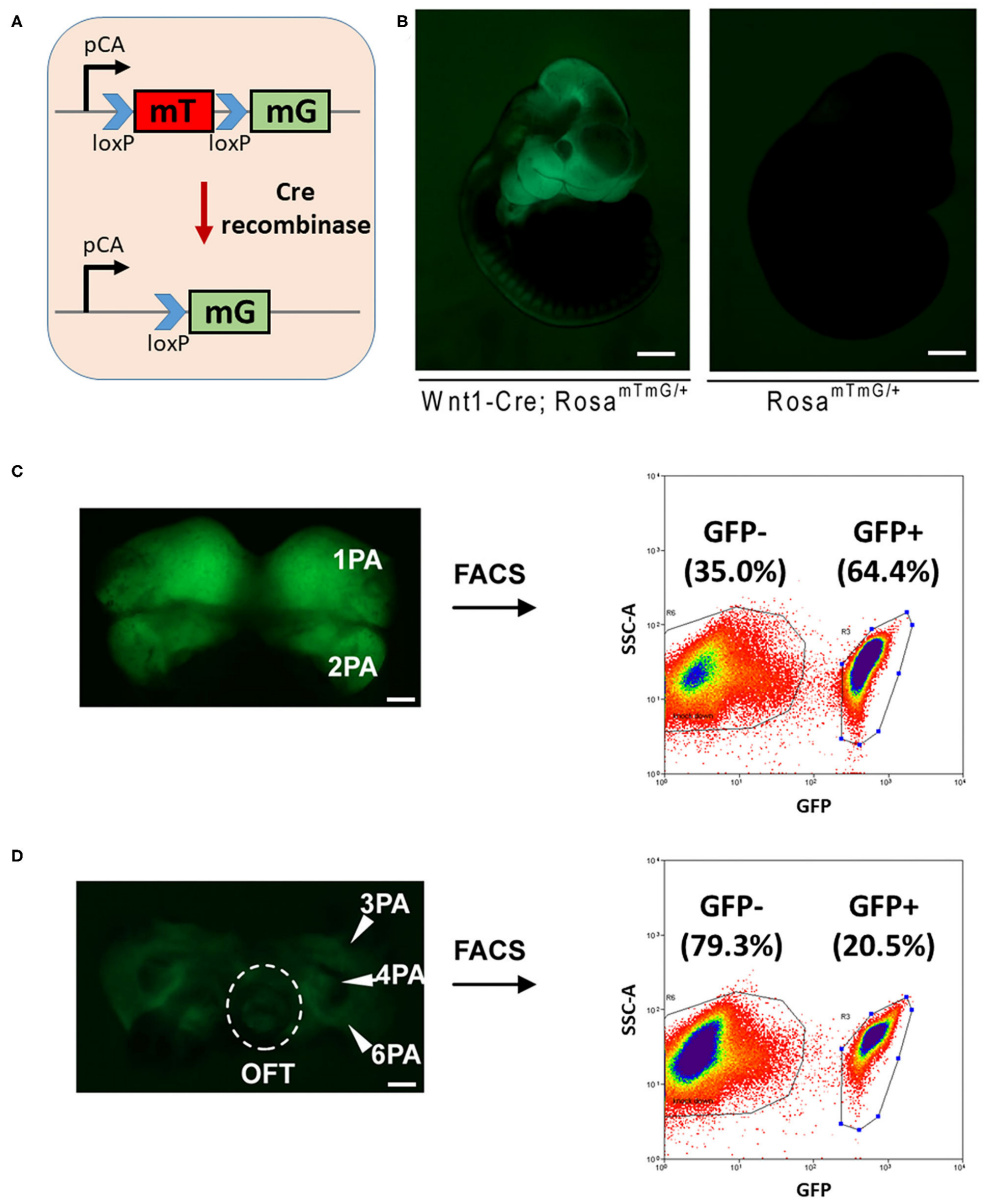
FACS sorting of NCCs from mouse embryos for miRNA profiling. **(A)** A schematic figure shows the *Rosa-mTmG* reporter line. **(B)** *Wnt1-Cre*-mediated tissue-specific expression of GFP in E10.5 embryos. Bar = 500 μm. The cranial-NCCs-derived tissue **(C)** and cardiac-NCCs-derived tissue **(D)** were dissected out, digested, and sorted for both GFP positive NCCs and GFP negative cells. Bar = 100 μm. PA, pharyngeal arch; OFT, outflow tract; FACS, fluorescence-activated cell sorting; NCCs neural crest cells; miRNA, microRNA; GFP, green fluorescent protein.

Cardiac NCCs, which are originated from the third, fourth, and sixth pharyngeal arches, populate the aorticpulmonary septum and conotruncal cushion and contribute to the smooth muscle of great vessels (1). Abnormal development in this group of cells often leads to ventricular septal defect and defects in great vessels connected to the heart (24). In order to study the potential interfering 3′UTR SNVs, which impair the regulation of miRNAs on their target mRNAs in CHD, we investigated the expression of miRNAs in cardiac NCCs. Rosa-mT-mG reporter mice (25), which possess *loxP* sites on either side of a membrane-targeted tdTomato (mT) cassette and express strong red fluorescence in all tissues and cell types, was bred with Wnt1-Cre mice (26). The presence of Cre recombinase will lead to the deletion of the mT cassette and the expression of the membrane-targeted GFP (mG) cassette located just downstream in NCCs (Figures 2A,B).

Embryonic (E) 10.5 Rosa-mT-mG/Wnt1-Cre GFP positive embryos were collected. The first and second pharyngeal arches were dissected out, digested, and sorted for both GFP positive cranial NCCs and GFP negative control cells by FACS sorting, and similarly, the third, fourth, and sixth pharyngeal arches and outflow tract were collected for cardiac NCCs. As a result, around 65 and 20% GFP positive cells were obtained from cranial-NCCs-derived (first and second pharyngeal arches) and cardiac-NCCs-derived (third, fourth, and sixth pharyngeal arches and outflow tract) tissues, respectively (Figures 2C,D). RNAs from both GFP positive cardiac NCCs and GFP negative non-NCCs were then subjected to profile the expression of miRNA using the approach of miRNA microarray.

As a result, a total of 201 and 221 miRNAs were detected expressed in GFP positive cardiac NCCs and GFP negative non-NCCs, respectively (Supplementary Table 1). miRNA members of several miRNA clusters, such as miR-17-92 cluster, miR-106a-363 cluster, and miR-106b-25 cluster, are highly expressed in GFP positive cardiac NCCs (Table 1). To our surprise, a similar list for the 30 most abundant miRNAs is found in GFP negative non-NCCs (Table 2). It is worthy to note that members of miR-17-92 clusters, including miR-17, miR-18a, miR-20a, miR-19b, and miR-92a, are highly expressed in both GFP positive and negative cell population. Next, we asked which miRNAs are enriched and which miRNAs have less expression in GFP positive cardiac NCCs when compared with GFP negative non-NCCs. Nine enriched miRNAs and seven less expressed miRNAs were identified with a cutoff in expression fold change (Log2 Fold change >1 or <-1) (Table 3).

**Table 1:** Top 30 most abundant miRNAs in cardiac NCCs.

**microRNAs**	**Relative expression in cardiac NCCs**	**Sequence**	**Cluster**
miR-709	13.275	GGAGGCAGAGGCAGGAGGA	Not in cluster
miR-17-5p	12.214	CAAAGUGCUUACAGUGCAGGUAG	miR-17-92 cluster
miR-20a-5p	11.196	UAAAGUGCUUAUAGUGCAGGUAG	miR-17-92 cluster
miR-92a-3p	10.689	UAUUGCACUUGUCCCGGCCUG	miR-17-92 cluster
miR-690	10.651	AAAGGCUAGGCUCACAACCAAA	Not in cluster
miR-106a-5p	10.517	CAAAGUGCUAACAGUGCAGGUAG	miR-106a-363 cluster
miR-93-5p	10.374	CAAAGUGCUGUUCGUGCAGGUAG	miR-106b-25 cluster
miR-103-3p	10.313	AGCAGCAUUGUACAGGGCUAUGA	Not in cluster
miR-214-3p	10.203	ACAGCAGGCACAGACAGGCAGU	miR-199a-214 cluster
miR-18a-5p	10.201	UAAGGUGCAUCUAGUGCAGAUAG	miR-17-92 cluster
miR-99b-5p	10.120	CACCCGUAGAACCGACCUUGCG	miR-99b-125a cluster
miR-16-5p	9.909	UAGCAGCACGUAAAUAUUGGCG	miR-15-16 cluster
miR-20b-5p	9.805	CAAAGUGCUCAUAGUGCAGGUAG	miR-106a-363 cluster
miR-107-3p	9.764	AGCAGCAUUGUACAGGGCUAUCA	Not in cluster
miR-181a-5p	9.763	AACAUUCAACGCUGUCGGUGAGU	miR-181a/b cluster
miR-181b-5p	9.622	AACAUUCAUUGCUGUCGGUGGGU	miR-181a/b cluster
miR-130b-3p	9.573	CAGUGCAAUGAUGAAAGGGCAU	miR-130b-301b cluster
miR-19b-3p	9.526	UGUGCAAAUCCAUGCAAAACUGA	miR-17-92 cluster
miR-26a-5p	9.457	UUCAAGUAAUCCAGGAUAGGCU	Not in cluster
miR-199a-5p	9.437	CCCAGUGUUCAGACUACCUGUUC	miR-199a-214 cluster
miR-125a-5p	9.361	UCCCUGAGACCCUUUAACCUGUGA	miR-99b-125a cluster
miR-199a-3p	9.167	ACAGUAGUCUGCACAUUGGUUA	miR-199a-214 cluster
miR-125b-5p	9.158	UCCCUGAGACCCUAACUUGUGA	miR-99a-125b cluster
miR-199b-3p	9.150	ACAGUAGUCUGCACAUUGGUUA	miR-199b-3154 cluster
miR-130a-3p	9.132	CAGUGCAAUGUUAAAAGGGCAU	Not in cluster
miR-145a-5p	9.010	GUCCAGUUUUCCCAGGAAUCCCU	miR-143-145 cluster
let-7e-5p	8.949	UGAGGUAGGAGGUUGUAUAGUU	miR-99b-125a cluster
miR-106b-5p	8.858	UAAAGUGCUGACAGUGCAGAU	miR-106b-25 cluster
miR-541-5p	8.856	AAGGGAUUCUGAUGUUGGUCACACU	Mirg cluster
miR-1195	8.752	UGAGUUCGAGGCCAGCCUGCUCA	Not in cluster

*miRNA, microRNA; NCCs, neural crest cells*.

**Table 2:** Top 30 most abundant miRNAs in non-NCCs.

**microRNAs**	**Relative expression in non-NCCs**	**Sequence**	**Cluster**
miR-709	13.38065	GGAGGCAGAGGCAGGAGGA	Not in cluster
miR-17-5p	12.06354	CAAAGUGCUUACAGUGCAGGUAG	miR-17-92 cluster
miR-690	11.37133	AAAGGCUAGGCUCACAACCAAA	Not in cluster
miR-99b-5p	10.85722	CACCCGUAGAACCGACCUUGCG	miR-99b-125a cluster
miR-20a-5p	10.73113	UAAAGUGCUUAUAGUGCAGGUAG	miR-17-92 cluster
miR-92a-3p	10.66891	UAUUGCACUUGUCCCGGCCUG	miR-17-92 cluster
miR-93-5p	10.25503	CAAAGUGCUGUUCGUGCAGGUAG	miR-106b-25 cluster
miR-103-3p	10.16812	AGCAGCAUUGUACAGGGCUAUGA	Not in cluster
miR-182-5p	10.07562	UUUGGCAAUGGUAGAACUCACACCG	miR-182-183 cluster
miR-16-5p	10.03377	UAGCAGCACGUAAAUAUUGGCG	miR-15-16 cluster
miR-106a-5p	9.949529	CAAAGUGCUAACAGUGCAGGUAG	miR-106a-363 cluster
miR-125a-5p	9.923689	UCCCUGAGACCCUUUAACCUGUGA	miR-99b-125a cluster
miR-107-3p	9.916354	AGCAGCAUUGUACAGGGCUAUCA	Not in cluster
miR-26a-5p	9.854673	UUCAAGUAAUCCAGGAUAGGCU	Not in cluster
miR-127-3p	9.778021	UCGGAUCCGUCUGAGCUUGGCU	miR-136-431 cluster
miR-145a-5p	9.766548	GUCCAGUUUUCCCAGGAAUCCCU	miR-143-145 cluster
miR-541-5p	9.682595	AAGGGAUUCUGAUGUUGGUCACACU	Mirg cluster
miR-214-3p	9.590926	ACAGCAGGCACAGACAGGCAGU	miR-199a-214 cluster
miR-181b-5p	9.429933	AACAUUCAUUGCUGUCGGUGGGU	miR-181a/b cluster
miR-130b-3p	9.42875	CAGUGCAAUGAUGAAAGGGCAU	miR-130b-301b cluster
miR-23b-3p	9.420783	AUCACAUUGCCAGGGAUUACC	miR-23b-24 cluster
miR-181a-5p	9.411454	AACAUUCAACGCUGUCGGUGAGU	miR-181a/b cluster
miR-125b-5p	9.3161	UCCCUGAGACCCUAACUUGUGA	miR-99a-125b cluster
miR-24-3p	9.314174	UGGCUCAGUUCAGCAGGAACAG	miR-23b-24 cluster
miR-18a-5p	9.269115	UAAGGUGCAUCUAGUGCAGAUAG	miR-17-92 cluster
let-7e-5p	9.152258	UGAGGUAGGAGGUUGUAUAGUU	miR-99b-125a cluster
miR-762	9.083022	GGGGCUGGGGCCGGGACAGAGC	Not in cluster
miR-1195	9.081083	UGAGUUCGAGGCCAGCCUGCUCA	Not in cluster
miR-130a-3p	9.068733	CAGUGCAAUGUUAAAAGGGCAU	Not in cluster
miR-351-5p	9.066365	UCCCUGAGGAGCCCUUUGAGCCUG	miR-322-351 cluster

*miRNA, microRNA; NCCs, neural crest cells*.

**Table 3:** A list of differentially distributed miRNAs between cardiac NCCs and non-NCCs.

**microRNAs**	**Relative expression level in cardiac NCCs**	**Relative expression level in non-NCCs**	**Log2 fold change**
miR-363-3p	5.819	N.D.	N/A
miR-466f-3p	4.940	N.D.	N/A
miR-542-5p	4.983	N.D.	N/A
miR-125b-1-3p	5.203	N.D.	N/A
miR-214-5p	7.908	6.294	1.614
miR-199a-5p	9.437	7.979	1.459
miR-20b-5p	9.805	8.417	1.388
miR-500-3p	6.367	5.160	1.207
miR-199b-3p	9.150	8.032	1.118
miR-23b-3p	8.358	9.421	−1.062
miR-127-3p	8.636	9.778	−1.142
miR-329-3p	4.746	5.950	−1.204
miR-708-5p	5.460	6.693	−1.233
miR-126a-3p	6.073	7.600	−1.526
miR-31-5p	4.626	6.576	−1.951
miR-182-5p	5.056	10.076	−5.020

*miRNA, microRNA; NCCs, neural crest cells; N.D., not detected; N/A, not applied*.

### Prediction of Disease-Related SNVs in 3′UTR That Interfere With miRNA Function

**Figure 3:**
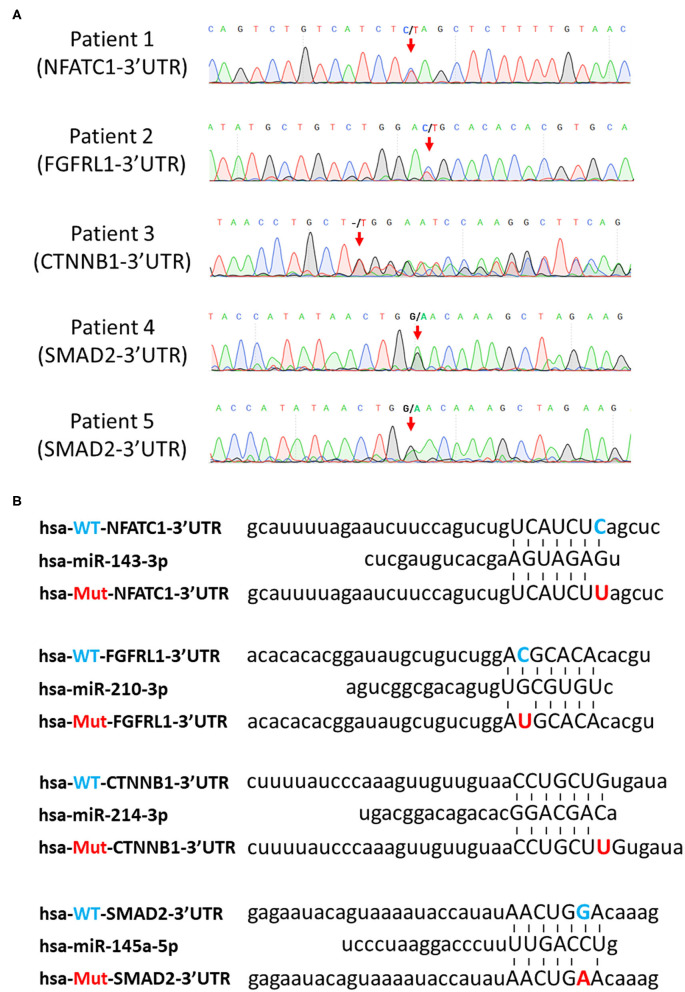
Prediction of SNV-mediated miRNA mis-regulating on their downstream targets. **(A)** Confirmation of selected SNVs by Sanger sequencing. Red arrows indicate the SNVs. **(B)** Prediction of pairing between the seed sequence of miRNAs and the SNV-involved mRNA 3′UTR regions. Blue letters indicate wild-type nucleotides, and red letters indicate mutant nucleotides. SNV, single-nucleotide variant; miRNA, microRNA.

Our previous study demonstrated that disruption of the expression of miRNA in NCCs leads to developmental defects in the heart and great arteries, which resemble defects observed in CHD patients (14). It indicated that miRNAs play critical roles in regulating cardiac NCCs in cardiac development. Therefore, we wonder whether certain SNVs identified above in 3′UTR connect to CHDs by interfering with the regulatory function of miRNA on gene expression. Since the seed sequence (nucleotides 2–8) of miRNA has been demonstrated to be critical to the pairing between miRNA and mRNA 3′UTR (27), we first searched miRDB (28), a miRNA target prediction database, for all predictions of miRNA-3′UTR pairing, in which the SNV is located in the sequence of 3′UTR pairing with seed sequence of miRNA. A total of 802 SNVs were identified being involved in such parings. The expression of miRNA in cardiac NCCs is the prerequisite of their regulatory function on gene expression. Next, we excluded the predictions associated with miRNAs not detected in our miRNA profiling in cardiac NCCs. Fifty-nine predictions remain in the list after selection. In the last step, we asked whether the remaining 59 SNVs were tightly associated with heart defects found in patients. As a result, four SNV candidates, NFATC1 c.^*^654C>T, FGFRL1 c.^*^414C>T, CTNNB1 c.^*^729_^*^730insT, and SMAD2 c.^*^7061G>A (Figure 3A), were identified in the analyses. miR-143-3p was predicted to mediate the c.^*^654C>T-induced dysregulation of transcriptional factor, NFATC1; the predictive regulation of fibroblast growth factor receptor like 1 by miR-210-3p is likely interfered by the mutation of c.^*^414C>T; an insertion of single thymine in the 3′UTR was predicted to lead to the mis-regulating of WNT signaling coactivator, beta-catenin (CTNNB1), by miR-214-3p; and Smad2, a key TGFβ signaling effector, was predicted to be mis-regulated by miR-145-5p because of the c.^*^7061G>A mutation (Figure 3B).

All these four mutations are rare SNVs. Surprisingly, all individuals with these SNVs are associated with conotruncal defect or septal defect based on the classification of CHD defects (Table 4), indicating these SNVs are likely causative mutations for congenital cardiac defects. Moreover, cardiac NCCs contribute to the development of conotruncus and ventricular septa; the observation of conotruncal or septal defects suggests the developmental defects of cardiac NCCs in these SNV-harbored patients. Since the pairing between mRNA 3′UTR and the seed sequence of miRNA is critical to the regulatory function of miRNA on gene regulation, the pairing between mutant 3′UTRs and miRNAs (Figure 3B) indicates that identified SNVs could induce the dys-regulation of NFATC1, FGFRL1, CTNNB1, and SMAD2 and further disturb the normal development of cardiac NCCs, which leads to the cardiac defects in patients.

**Table 4:** The distribution of four rare SNVs in control and case groups.

**SNVs**	**Number in control group**	**Number in case group**	**Defects**
NFATC1 c.*654C>T	0	1	Conotruncal (1)
FGFRL1 c.*414C>T	0	1	Conotruncal (1)
CTNNB1 c.*729_*730insT	0	1	Conotruncal (1)
SMAD2 c.*7061G>A	0	2	Conotruncal (1); septal (1)

*SNV, single-nucleotide variant*.

### Experimental Validation of miRNA-Mediated Gene Dysregulation Induced by SNVs

**Figure 4:**
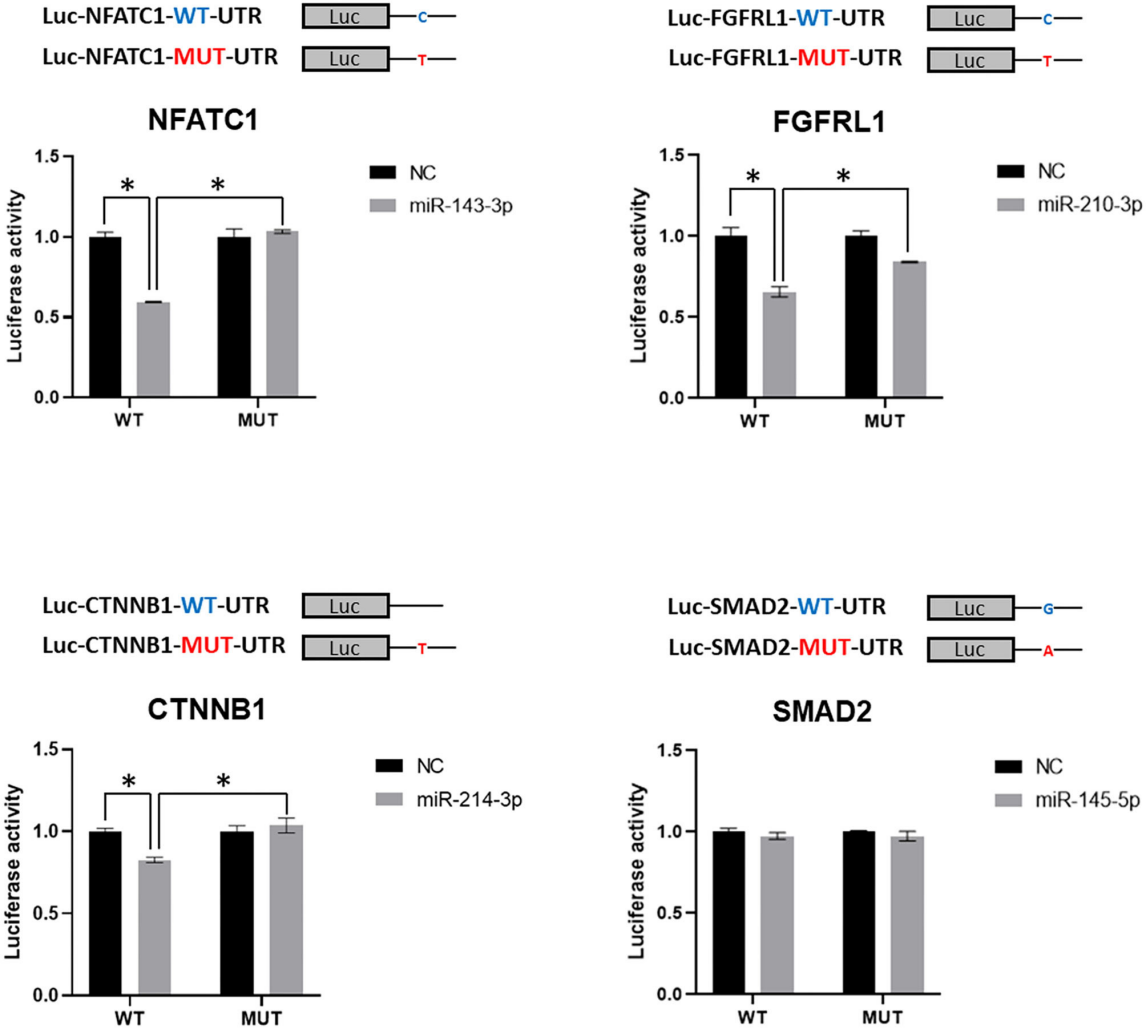
Validation of miRNA-mediated dysregulation induced by SNVs. Luciferase reporter assay was applied in the validation. Luciferase reporters with wild-type or mutant 3′UTR of selected genes were co-transfected with predicted miRNA mimics, which was predicted to target the tested 3′UTR of gene, or negative control (NC), and luciferase activity was determined. Values are presented as relative luciferase activity ± SD relative to the luciferase activity of reporter co-transfected with negative control mimics. Data were obtained from at least three independent experiments. miRNA, microRNA; SNV, single-nucleotide variant. *indicates *p* < 0.05.

To further confirm the miRNA-mediated gene dysregulation induced by SNVs, wild-type, and mutant 3′UTRs, which harbor the same point mutations as detected SNVs, were cloned into the 3′UTR region of luciferase reporter. The predicted regulatory miRNA was then tested for the repressive effect on luciferase activity of wild-type and mutant reporters in a luciferase assay. As shown in Figure 4, miR-143-3p, miR-210-3p, and miR-214-3p inhibited the activities of wild-type Luc-NFATC1-WT-UTR, Luc-FGFR1-WT-UTR, and Luc-CTNNB1-WT-UTR reporters, respectively, which is consistent with the prediction. However, miR-145-5p, which was predicted to bind to 3′UTR of SMAD2, showed no repression on wild-type Luc-SMAD2-WT-UTR reporter. More importantly, the repressive effects of miR-143-3p, miR-210-3p, and miR-214-3p on NFATC1, FGFR1, and CTNNB1 3′UTR luciferase reporters were diminished when the identified SNV was introduced (Figure 4). It is worthy to note that the mutation of NFATC1 c.^*^654C>T and CTNNB1 c.^*^729_^*^730insT, which similarly disrupts the pairing between the second nucleotide of miRNA (the first nucleotide of the miRNA seed sequence) and 3′UTR, totally abolished the regulatory function of miR-143-3p on NFATC1 and miR-214-3p on CTNNB1, respectively. Therefore, our data confirmed that three CHD-associated SNVs identified in this study, NFATC1 c.^*^654C>T, FGFRL1 c.^*^414C>T, and CTNNB1 c.^*^729_^*^730insT, induce the miRNA-mediated gene dysregulation.

## Discussion

The etiology of congenital heart disease is complex. Although environmental factors are involved in the pathogenesis of CHDs, genetic mutation—both inherited mutation and de novo mutation—is the major cause of CHDs. Huge efforts have been spent on investigating the genetic cause responsible for the CHDs we observed, and some mutations have been successfully linked to syndromes associated with CHDs. For example, the deletion of 3 million base pairs on one copy of chromosome 22 results in DiGeorge syndrome in human patients (29). Genetic studies have linked ~50% of Noonan syndrome to mutations in PTPN11 gene, which encodes SHP-2 protein, an SH2 domain-containing, non-receptor tyrosine phosphatase (PTPase) essential for cellular proliferation, differentiation, and migration (30). However, many CHD cases without apparent linkage to environmental interfering remains idiopathic. miRNAs have been demonstrated to be closely associated with different human diseases, such as cancer, cardiovascular diseases, hepatitis, diabetes, etc. (31). It is not surprising that miRNAs are involved in the pathogenesis of CHDs, which is supported by our previous report (14). In this study, we further showed that three CHD-associated SNVs in 3′UTR could lead to the miRNA-mediated gene dysregulation. More cases should be involved in the future to demonstrate how important this type of mutation is in the cause of human CHDs. Previous studies have shown that precise control of key signaling cascade activity is critical to proper embryonic development (32), including the induction of neural crest (33). Disease-associated SNVs in 3′UTR of these tightly controlled genes could break the balance due to the dysregulation by miRNAs even though the mutation is only present in one allele, which results in the situation that 50% of mRNAs for one gene are mutant in theory and have abnormal miRNA-mediated regulation. The detailed mechanism of this miRNA-mediated gene dysregulation in cardiac developmental defects needs to be further investigated *in vivo*. For example, mutations identified from patients in this study should be introduced in a mouse model to further confirm their CHD-causative effect, and the miRNA-mediated molecular mechanism of CHD should be further validated with these genetically engineered mouse embryo *in vivo* as well.

Several studies indicated that SNVs in human CHD patients could lead to miRNA-mediated gene dysregulation. For example, +1,905G>A in 3′UTR region of methionine synthase (MTR) gene, which is associated with CHDs, was reported to result in dysregulation of MTR by miR-485, miR-608, and miR-1293 (34). Point mutation of TBX5 3′UTR sequence, TBX5 c.^*^1101C4T, is closely associated with septation defects in human patients and leads to miR-9 and miR-30a-mediated dysregulation of TBX5 expression (35). The expression of these miRNAs during cardiac development is not well-characterized. The majority of conotruncal defect and ventricular septal defect are very likely linked to the defect of cardiac NCCs. The migration and differentiation of cardiac NCCs is transient and happens in the early developmental stage of the embryo. Therefore, it is challenging to obtain cardiac NCCs from the human embryo. Due to the high conservation of mammalian embryo development, we obtained the expression profile of miRNAs from mouse cardiac NCCs and focused on the miRNAs with high expression level in this study. Three rare SNVs were identified with the great potential of disrupting the normal miRNA-mediated gene regulation in our study. More importantly, these SNVs are all associated with cardiac NCCs-involved conotruncal defect and septal defect in human patients. Therefore, our study indicates that miRNA-mediated gene dysregulation in cardiac NCCs could be an important etiology of congenital heart disease.

Due to the limitation of our sequencing strategy, only ~250 protein-coding genes were studied in this study, although most of them are considered important genes for cardiac development. Since protein-coding genes are widely regulated by miRNAs (27), more genes, if not all, should be investigated with the whole genome sequencing data in the future. Our study provides a cue that miRNA-involved gene regulation might be important but overlooked in the etiological study of human congenital heart disease. Mutations of miRNA coding sequence and sequence regulating the transcription of miRNA primary transcript or the processing of miRNA precursor warrant further investigation in human patients with CHD.

## Data Availability Statement

The datasets presented in this study can be found in online repositories. The names of the repository/repositories and accession number(s) can be found below: NCBI (accession: GSE178823).

## Ethics Statement

The studies involving human participants were reviewed and approved by the medical ethics committee of Children's Hospital of Fudan University. Written informed consent to participate in this study was provided by the participants' legal guardian/next of kin. The animal study was reviewed and approved by the medical ethics committee of the First Affiliated Hospital, Sun Yet-sen University.

## Author Contributions

WL, LC, KC, JW, and Z-PH prepared the manuscript. WL, LC, RP, and Y-LT analyzed the clinical and sequencing data from the cohort. KC performed related bioinformatic analyses. JW performed cloning experiments and luciferase assay. JC provided sorted NCCs from mouse embryo. YY supervised the process of bioinformatic analyses. PL supervised the analyses of clinical and sequencing data for the cohort. Z-PH drafted the final version of the manuscript. All authors read and approved the final manuscript.

## Funding

This work was supported by grants from the Guangdong Science and Technology Department (2018A050506026 to Z-PH and 2019B030316024), the Science and Technology Planning Project of Guangzhou, China (202002020064 to WL), the China Postdoctoral Science Foundation (2020M672981 to LC), the National Natural Science Foundation of China (81873463 to Z-PH and 81970227 to JC), the Guangdong Basic and Applied Basic Research Foundation (2019B151502003 to Z-PH), the Fundamental Research Funds for the Central Universities (20ykzd06 to Z-PH), and the donation for scientific research from the Terry Fox Foundation.

## Conflict of Interest

The authors declare that the research was conducted in the absence of any commercial or financial relationships that could be construed as a potential conflict of interest.

## Publisher's Note

All claims expressed in this article are solely those of the authors and do not necessarily represent those of their affiliated organizations, or those of the publisher, the editors and the reviewers. Any product that may be evaluated in this article, or claim that may be made by its manufacturer, is not guaranteed or endorsed by the publisher.
