# Supplementary figures and images for: Identification of Novel Single-Nucleotide Variants With Potential of Mediating Malfunction of MicroRNA in Congenital Heart Disease
Source: Front Cardiovasc Med. 2021 Sep 10;8:739598. doi: 10.3389/fcvm.2021.739598 (PMC8460875; doi:10.3389/fcvm.2021.739598)

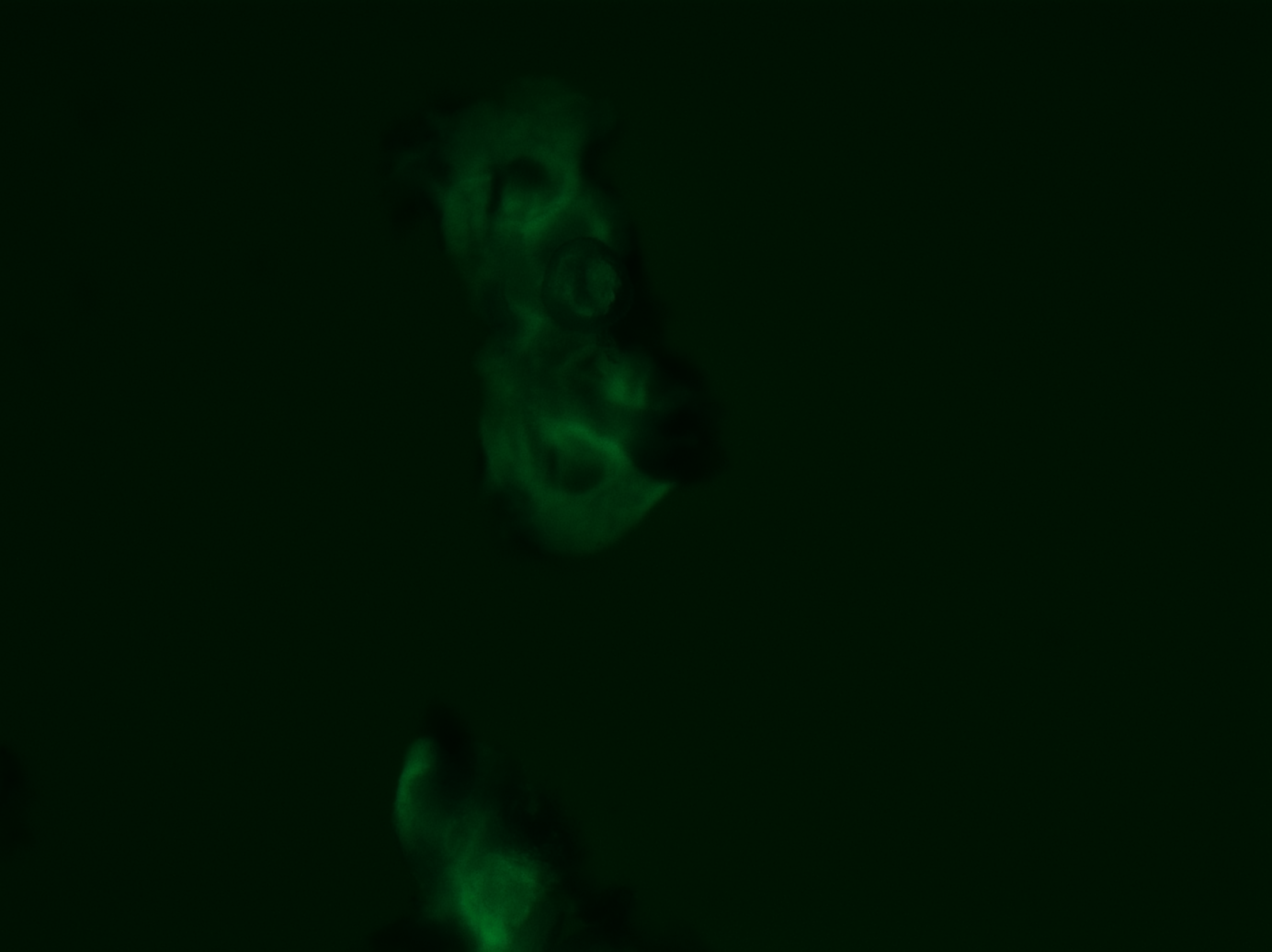

Supplement: Supplementary file 2 [file Data_Sheet_1.ZIP › image for dissection of cardiac neural crest derivatives.tif]

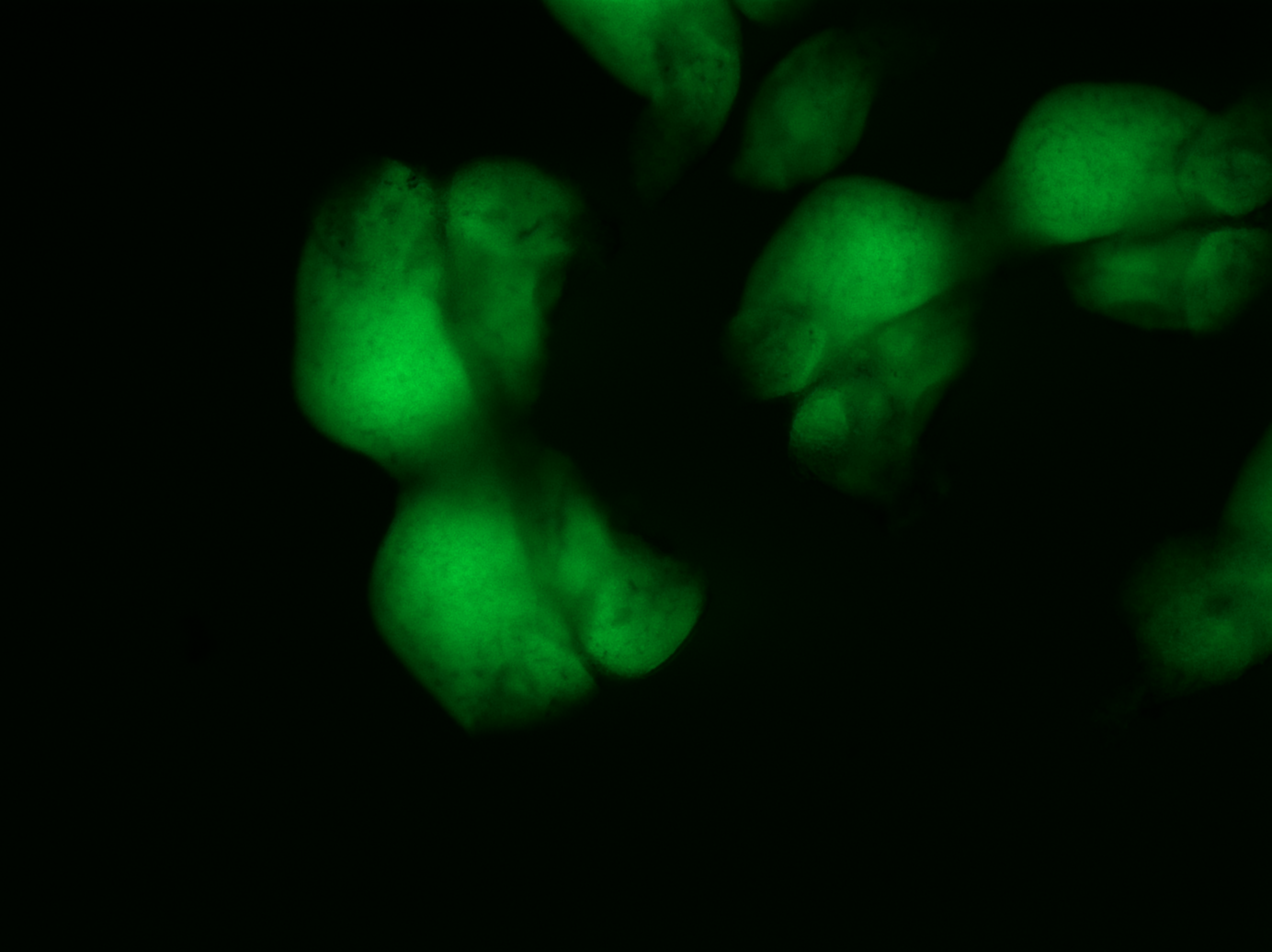

Supplement: Supplementary file 2 [file Data_Sheet_1.ZIP › image for dissection of cranail neural crest derivatives.tif]

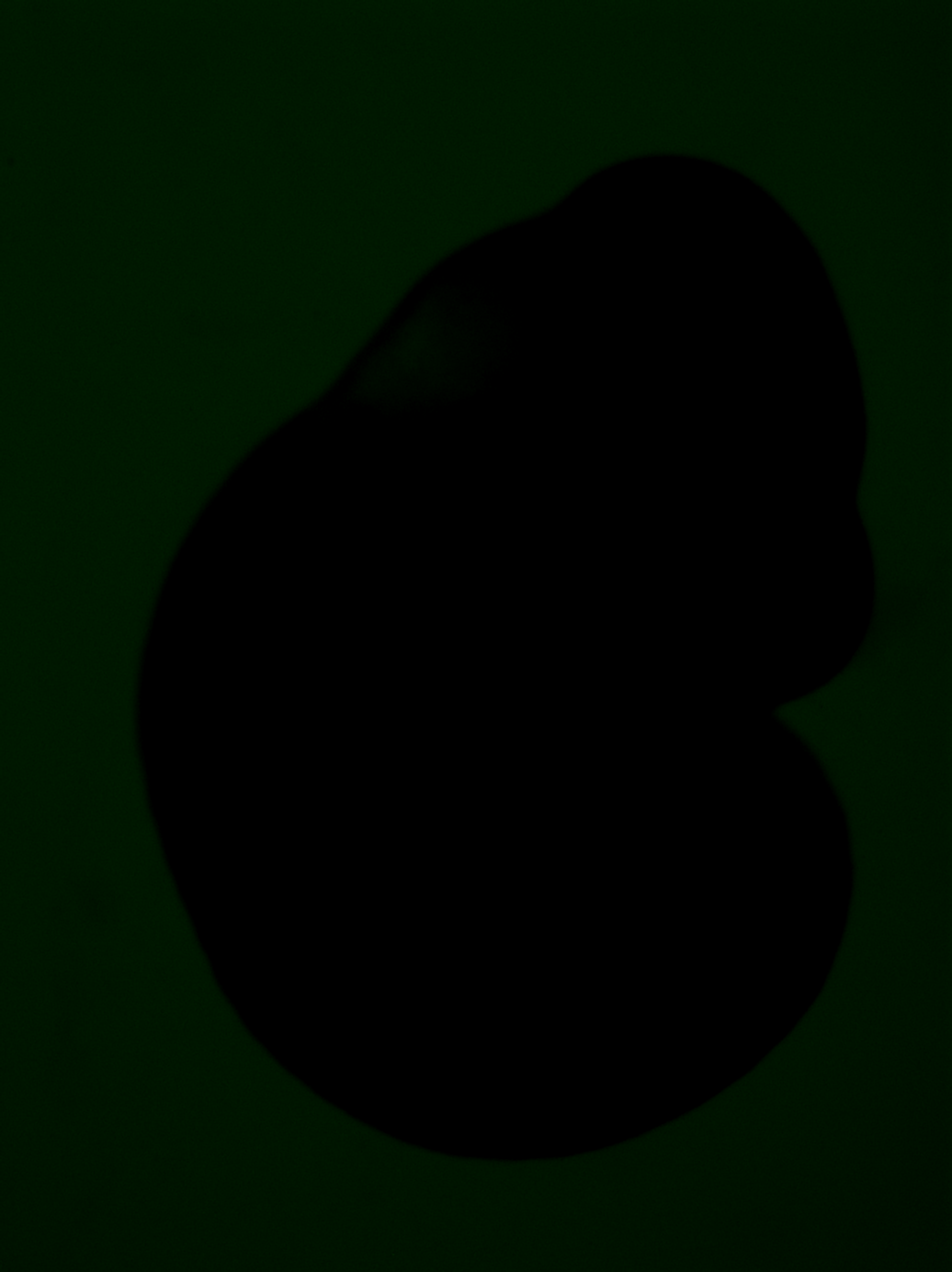

Supplement: Supplementary file 2 [file Data_Sheet_1.ZIP › image for Rosa-only embryo.tif]

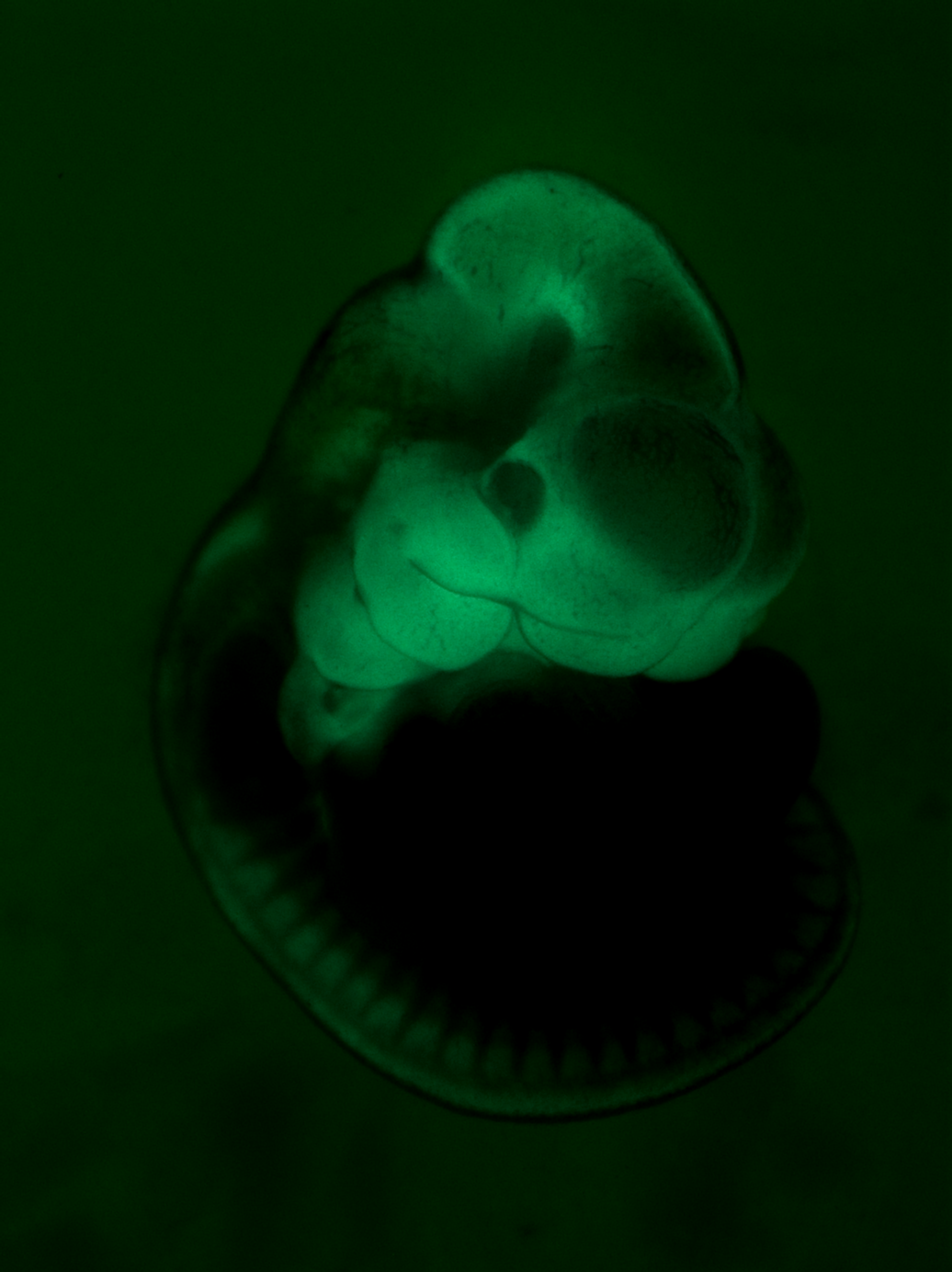

Supplement: Supplementary file 2 [file Data_Sheet_1.ZIP › image for Wnt1-Cre-Rosa embryo.tif]
